# Targeting PI3K/Akt/mTOR Pathway by Different Flavonoids: A Cancer Chemopreventive Approach

**DOI:** 10.3390/ijms222212455

**Published:** 2021-11-18

**Authors:** Torki A. Zughaibi, Mohd Suhail, Mohammad Tarique, Shams Tabrez

**Affiliations:** 1 King Fahd Medical Research Center, King Abdulaziz University, Jeddah 21589, Saudi Arabia; taalzughaibi@kau.edu.sa; 2 Department of Medical Laboratory Technology, Faculty of Applied Medical Sciences, King Abdulaziz University, Jeddah 21589, Saudi Arabia; 3 Department of Child Health, School of Medicine, University of Missouri, Columbia, MO 65201, USA; tariqueunmatched@gmail.com

**Keywords:** Akt, cancer, flavonoids, inhibitors, mTOR, PI3K

## Abstract

Cancer is, globally, one of the main causes of death. Even though various therapies are available, they are still painful because of their adverse side effects. Available treatments frequently fail due to unpromising responses, resistance to classical anticancer drugs, radiation therapy, chemotherapy, and low accessibility to tumor tissues. Developing novel strategies to minimize adverse side effects, improve chemotherapy sensitivity, and control cancer progression is needed. Many studies have suggested small dietary molecules as complementary treatments for cancer patients. Different components of herbal/edible plants, known as flavonoids, have recently garnered attention due to their broad biological properties (e.g., antioxidant, antiviral, antimicrobial, anti-inflammatory, anti-mutagenic, anticancer, hepatoprotective, and cardioprotective). These flavonoids have shown anticancer activity by affecting different signaling cascades. This article summarizes the key progress made in this area and discusses the role of flavonoids by specifically inhibiting the PI3K/Akt/mTOR pathway in various cancers.

## 1. Introduction

Cancer is a group of diseases where cells grow uncontrollably, and abnormal cells spread throughout the body via the bloodstream and the lymphatic system [1]. According to the World Health Organization (WHO), cancer was the second most lethal disease in 2019 [2]. Recently, a GLOBOCAN report estimated that there were approximately 10 million deaths due to cancer and 19.3 million new cases in 2020 [3]. Furthermore, a report published by WHO on 4 February 2020, warned that if the current upward trend in cancer incidences continues, the world will see a 60% rise in cancer cases in the next 20 years [2]. There are many reasons for the occurrence of cancer, but one possible cause is the aberrant regulation of different cell signaling pathways due to the acquisition of genetic and epigenetic changes [4]. One such pathway is the phosphoinositide 3-kinase (PI3K)-protein kinase B (Akt)-mammalian target of rapamycin (mTOR). Several studies have reported the inappropriate PI3K/Akt/mTOR pathway regulation in different cancers, such as breast, liver, colorectal, prostate, and gastric cancer [5,6,7]. Hence, the PI3K/Akt/mTOR pathway has become a “hot spot” of molecular biomarker-based/targeted therapy of different tumors.

**Figure 1:**
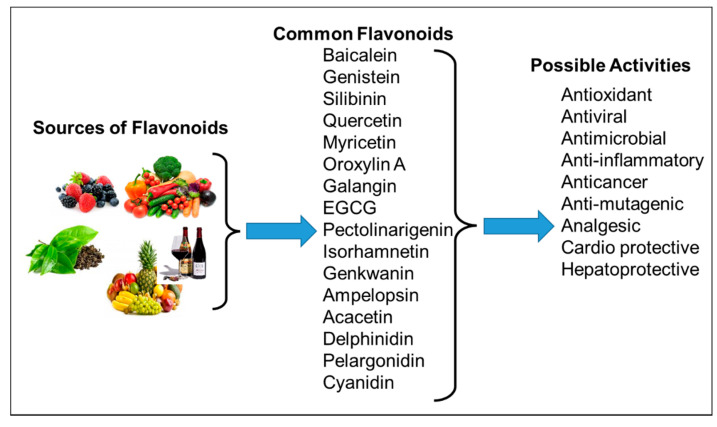
Common flavonoids from dietary sources, with their biological activities. EGCG; Epigallocatechin gallate. (Source: The effects of polyphenols and other bioactives on human health. https://pubs.rsc.org/image/article/2019/fo/c8fo01997e/c8fo01997e-f1_hi-res.gif accessed on 29 October 2021).

Natural compounds obtained from plant sources have recently garnered interest due to their easy availability, non-toxic/low adverse effects, cost-effectiveness, and ability to modulate multiple pathways [8]. Among the natural compounds, flavonoids have gained attention as anticancer agents, and are documented as being effective against various types of cancer [9,10]. Flavonoids are of low-molecular-weight, comprising polyphenolic compounds, classified into six groups—isoflavonoids, flavanones, flavanols, flavonols, flavones, and anthocyanidins [11]. The primary source of these flavonoids is the regular human diet, including fruits, vegetables, grains, bark, roots, stems, flowers (Table 1 and Figure 1), plant-derived beverages, such as green tea, wine, and cocoa-based products [12,13,14,15,16,17,18,19,20]. Flavonoids have shown various activities, such as inhibiting cell proliferation and angiogenesis, cell cycle arrest, induction in apoptosis, and reversion in multidrug resistance [21,22]. Furthermore, it has also been reported to act as a pro-oxidant in some cases, and may interact with other therapeutic agents during biotransformation [23]. Rapid metabolism, low solubility, and poor absorption in the gastrointestinal tract hinder the real pharmacological potential of dietary flavonoids [24].

## 2. The Implication of PI3K/Akt/mTOR Pathway in Cancer

The PI3K/Akt/mTOR pathway is one of the most deregulated signaling cascades involved in the development of different human cancers. Each central node of this pathway is highly activated in most tumors [39,40]. The central nodes include phosphatidylinositol 4,5-bisphosphate 3-kinase catalytic subunit alpha (PIK3CA), receptor tyrosine kinase (RTK) class I (Epidermal growth factor receptor; EGFR, human epidermal growth factor receptor 2; HER2, etc.), Akt, and phosphatase and tensin homolog deleted on chromosome 10 (PTEN). The *PIK3CA* gene encoding p110α catalytic subunit of PI3K is often mutated in most of cancer types [41,42]. Mutation in *PIK3CA* and independent activation of the PI3K pathway only (without Akt) can also induce cancer [43,44]. On the other hand, a mutation in the *EGFR* gene acts as an activator of PI3K and plays a role in the pathogenesis of non-small cell lung cancer [45]. Similarly, overexpression and amplification of the *EGFR* gene are frequently observed in glioblastoma [46]. Another member of the EGFR family, *HER2*, is overexpressed and amplified in invasive gastric and breast cancers. However, its overexpression is less frequently observed in other cancer types, such as ovarian, colon, salivary, biliary, and lung cancer [47]. The somatic mutations and amplification in pleckstrin homology (PH) domain (E17K) of Akt1 have been identified in various cancers, such as pancreatic, colorectal, and ovarian, and breast cancers [48]. The PI3K/Akt/mTOR pathway is a master regulator of cancer progression and is considered as one of the most important therapeutic targets. The PI3Ks phosphorylate phosphatidylinositol 4,5-bisphosphate (PIP2) to phosphatidylinositol-3,4,5-triphosphate (PIP3), leading to Akt phosphorylation that affects the cancer cell growth, cell survival, and cell cycle [49,50]. At the same time, phosphatase, and tensin homolog (PTEN) act as antagonists of PI3K and dephosphorylate PIP3 into PIP2 [51,52]. The complete blockage of PI3K signaling might effectively control the progression of different types of cancer [50,53].

**Figure 2:**
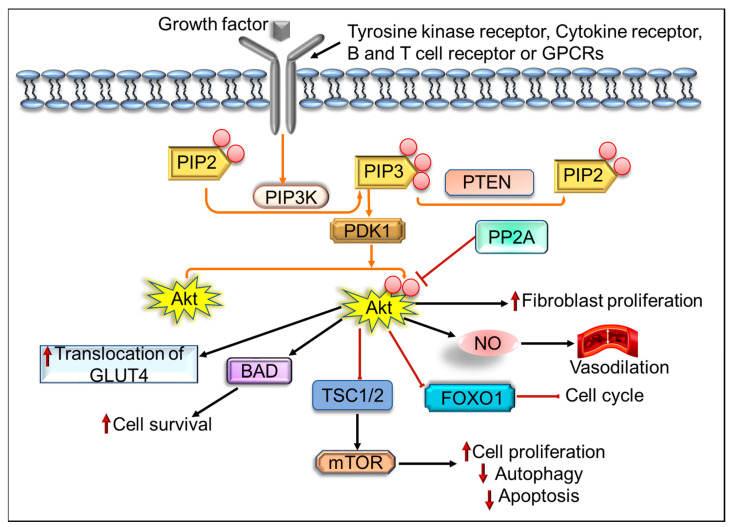
PI3K/Akt signaling pathway; PIP2, phosphatidylinositol 4, 5-bisphosphate; PTEN, phosphatase and tensin homolog deleted on chromosome 10; PDK1, 3-phosphoinositide-dependent kinase 1; PP2A, protein phosphatase 2A; BAD, BCL2 associated agonist of cell death; GLUT4, glucose transporter type 4; GPCRs, G-protein-coupled receptors; TSC, tuberous sclerosis complex; FOXO1, forkhead box O1 protein; NO, nitric oxide.

Akt plays an important role in regulating tumor-associated cell processes, including cell survival, growth, migration, cell cycle progression, angiogenesis, and epithelial-mesenchymal transition [54]. Inhibition of the Akt pathway induces apoptosis and inhibits Akt-associated tumor cell growth [55,56]. The activation of the Akt pathway takes place through different receptors, such as integrin receptors, cytokine receptors, B and T cell receptors, tyrosine kinases receptor, and G-protein-coupled receptors (GPCRs) (Figure 2) through PIP3 generated by PI3Ks [57,58]. PIP3 does not activate Akt directly but modifies Akt configuration by binding to its PH domain and recruit Akt to the plasma membrane allowing phosphoinositide-dependent kinase-1 (PDK1) to phosphorylate the kinase domain at Thr308 residue [59,60]. The activated Akt leads to the phosphorylation of different downstream proteins present in the nucleus, cytosol, plasma membrane, supporting cell growth and survival, among other cellular effects [61]. On the other hand, dephosphorylation of Akt at Thr308 and Ser473 residues, by protein phosphatase 2A (PP2A), leads to its inhibition [62], and could increase fibroblast proliferation, vasodilatation, inhibition of the forkhead box O1 (FOXO1) protein, cell cycle arrest, and activation of B-cell lymphoma 2 (Bcl-2) associated agonist of cell death (BAD), leading to increased cell survival, stimulation of mTOR, resulting in reduced apoptosis and autophagy, and increased translocation of glucose transporter type 4 (GLUT4) [63]. Several scientific reports suggested an aberrant Akt signaling pathway in different types of cancer, resulting in tumor aggressiveness in some cases. Abnormalities in *Akt* genes have been reported in various human cancers, such as gastric carcinoma, glioblastoma, and gliosarcoma, whereas Akt2 amplification has been reported in head and neck squamous cell carcinoma, pancreatic, ovarian, and breast cancers [64].

The mTOR pathway also plays a vital role in regulating different activities, such as cell survival, cell growth, metabolism, and protein synthesis in response to upstream signals [65]. This is a downstream substrate of PI3K and Akt with two distinct complexes mTORC1 and mTORC2 [66]. Akt activates mTOR activity either by direct phosphorylation of mTOR at Ser2448 or by indirect phosphorylation and inhibition of tuberous sclerosis complex 2 (TSC2). Direct phosphorylation of TSC2 at S939 and T1462 [67,68] by Akt releases its inhibitory effect on mTOR and upregulates mTOR activity. TSC2 makes a heterodimeric complex with TSC1 and acts as a negative regulator of GTPase-activating protein (GAP) activity [69]. Because TSC2 suppresses the activity of the Ras-related GTPase Rheb, a selective activator of mTORC1, inhibition of TSC2 by Akt results in activation of mTORC1 [70]. The hyperactivation of this cascade can stimulate tumor development and progression through different mechanisms such as promoting growth factor receptor signaling, suppression of autophagy, lipid metabolism, glycolytic metabolism, angiogenesis, and cancer cell migration [71,72]. The different growth factors, such as vascular endothelial growth factor, hepatocyte growth factor, transforming growth factor, platelet-derived growth factor, insulin-like growth factor 1, and epidermal growth factor regulate the activity of mTOR signaling [73].

## 3. Inhibition of PI3K/Akt/mTOR Signaling Pathway by Different Flavonoids

**Figure 3:**
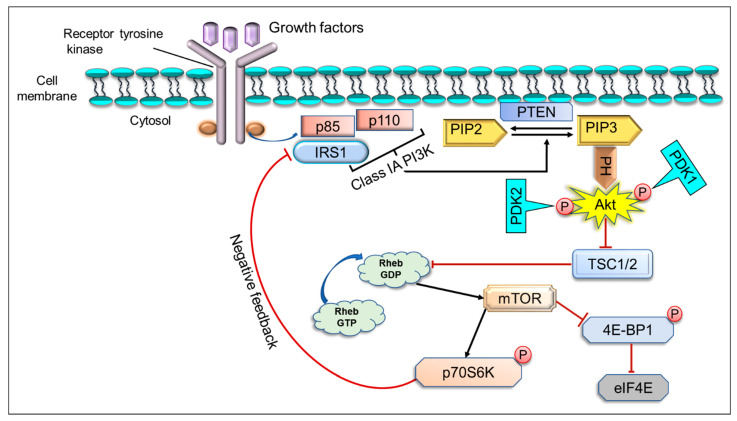
mTOR signaling cascade; IGF-1, insulin-like growth factor 1; EGF, epidermal growth factor; TGF, transforming growth factor; VEGF, vascular endothelial growth factor; PIP2, phosphatidylinositol 4, 5-bisphosphate; PDK1, 3-phosphoinositide-dependent kinase 1; TSC1/2, tuberous sclerosis complex 1/2; mTOR, mammalian target of rapamycin; PI3K, phosphatidylinositol 3-kinase; eIF4E, eukaryotic translation initiation factor 4E; PTEN, phosphatase and tensin homolog deleted on chromosome 10; IRS1, insulin receptor substrate 1; 4EBP1, eukaryotic translation initiation factor 4E-binding protein 1; p70S6K1, p70 ribosomal S6 kinase 1; Rheb GDP, Ras homolog enriched in brain GDP; Rheb GTP, Ras homolog enriched in brain GTP.

PI3K/Akt/mTOR signaling pathways are crucial to multiple aspects of cell growth and survival in physiological and pathological conditions, such as cancer [74]. In response to extracellular stimuli, the recruitment of class IA PI3K to the plasma membrane occurs by interaction of p85 and insulin receptor substrate (IRS) through the activation of RTKs or GPCRs [75]. The heterodimeric class IA PI3Ks phosphorylate PIP2 at position 3 of the inositol ring to convert it into PIP3, which acts as a second cellular messenger that controls cell growth, cell survival, and proliferation [76,77,78]. PIP3 binds to the PH domain of Akt and translocates it to the plasma membrane (Figure 3), where PDK-1 phosphorylates Akt [60,79]. Once Akt is activated, it further phosphorylates a broad array of proteins involved in cell cycle regulation, growth, proliferation, apoptosis, and cell survival [63,80]. The phosphatase PTEN plays a negative modulator of mTOR cascade [81]. It inhibits the signaling through the PI3K-Akt pathway through the involvement of TSC1/2 [82]. Deregulation of various components of the mTOR pathway, such as PI3K amplification/mutation, loss of PTEN function, overexpression of Akt, ribosomal protein S6 kinase beta-1 (S6K1), eukaryotic translation initiation factor 4E binding protein 1 (4EBP1), and overexpression of eukaryotic translation initiation factor 4E (eIF4E), has been reported in numerous cancers, especially melanoma, where variation in key elements of the mTOR signaling have major effects on tumor growth [83]. One study suggested natural compounds and herbs, such as resveratrol, diosgenin, timosaponin III, 3,3’-diindolylmethane, epigallocatechin gallate (EGCC), pomegranate, curcumin, gallic acid, and genistein, could directly or indirectly inhibit the mTOR pathway [84]. In the below-mentioned section, we have listed some well-known flavonoids reported as anticancer agents in various cancer models (Table 2).

### 3.1. Quercetin

Quercetin is a flavonol and is a subclass of flavonoids. Some vegetables and fruits, such as onions, scallions, kale, broccoli, apples, berries (and even teas), are the primary sources of quercetin [23]. Some studies reported that quercetin inhibits phosphorylation of the mTOR primary downstream targets, namely 4E-BP1 and ribosomal protein S6K [101,102,103]. It has shown a potential anticancer activity in various cancer cell lines and animal models in a dose-dependent manner. Quercetin has been reported to be more cytotoxic compared to ellagic acid and it inhibits cell cycle progression in the S phase in leukemia and breast cancer cells. It has also shown to have a ~5-fold increase in the life span of tumor-bearing mice than untreated mice [104].

### 3.2. Myricetin

Myricetin, a plant-derived flavonoid, commonly exists in fruits and other foods/beverages, such as oranges, berries, nuts, tea, red wine, and vegetables (tomatoes) [105], possessing anticancer effects [28,106]. It inhibits cell cycle progression and proliferation and induces apoptosis and autophagy in human colon cancer cells by inhibiting the PI3K/Akt/mTOR signaling [107]. Myricetin also suppresses breast cancer cell growth and inhibits UVB-induced skin cancer [108,109]. One study reported that myricetin induces apoptosis through ROS induction and inhibits cell migration, tube formation, and PI3K/Akt/mTOR signaling in human umbilical vascular endothelial cells [28].

### 3.3. Kaempferol

Kaempferol is a natural flavonol commonly found in plants and fruits, such as kale, beans, green tea, Brussels sprouts, spinach, apple, grapefruit, and broccoli [110]. It has been reported to have antioxidant and antitumor properties. Kaempferol exerts strong anticancer effects through inducing apoptosis, cell migration, cell cycle arrest at the G2/M phase, inhibiting and reducing the level of mTOR, pm-TOR, PI3K, p-PI3K, and Akt protein levels in the human malignant melanoma A375 cell line [29]. Further, it exerts anti-proliferative effects on lung cancer and human endothelial cells by activating mitogen-activated protein kinase (MAPK) signaling [111]. A recent study also suggested potent anticancer, anti-proliferation activity of kaempferol in liver cancer [112]. In addition, kaempferol has been reported to significantly inhibit HepG2 cell proliferation, invasion, and migration, and induce apoptosis by up/downregulating PTEN and microRNA-21 (miR-21), respectively, ultimately inhibiting the PI3K/Akt/mTOR pathway [85].

### 3.4. Isorhamnetin

Flavonoid isorhamnetin obtained from the medicinal plant *Hippophae rhamnoides* L. has shown anticancer effects in colorectal cancer. It has been reported to suppress cell proliferation and induce the G2/M phase cell cycle arrest by inhibiting the PI3K/Akt/mTOR pathway in colorectal and breast cancer [33,86].

### 3.5. Green Tea Catechins, Epicatechin, and Epigallocatechin-3-Gallate

Green tea catechin, such as epicatechin and epigallocatechin-3-gallate, is present in green tea, a typical refreshment drink enjoyed worldwide [113]. Epigallocatechin-3-gallate has shown significant anticancer activities in different cancer models [114]. Recent studies have suggested that epicatechin interacts and neutralizes reactive oxygen species (ROS) in the cell and modulates the MAP kinase pathway to inhibit cell proliferation [115]. In addition, it has shown inhibitory activities against Akt and NF-κB in combination with panaxadiol or cisplatin in HCT-116 and renal tubular carcinoma [116]. Some evidence shows that it downregulates doxorubicin-induced overexpression of P-glycoprotein through the inhibition of PI3K/Akt and mitogen-activated protein kinase kinase/extracellular signal-regulated kinase (MEK/ERK) signaling pathways [117,118]. Additionally, it downregulates the PI3K/Akt and MEK/ERK signaling pathways and promote apoptosis in T47D cells of human breast cancer [119,120].

### 3.6. Fisetin

Fisetin is a flavonol commonly found in some fruits/plants, such as strawberries, grapes, apples, persimmons, onions, kiwi, kale, etc. It shares antioxidant properties with many other plant polyphenols [121]. A study reported that a dietary tetrahydroxyflavone, fisetin inhibited human non-small cell lung cancer cells by downregulating the PI3K/Akt/mTOR signaling pathway [122]. Fisetin has shown to downregulate the PTEN protein levels in multiple myeloma U266 cells and A549 lung carcinoma [122,123]. In addition, it reduces phosphorylation of Akt, mTOR, microphthalmia-associated transcription factor (MITF), and p70S6K proteins in human melanoma 451Lu cells in a dose-dependent manner [122,124].

### 3.7. Lupiwighteone

Isoflavone, lupiwighteone is majorly present in medicinal plants *Glycyrrhiza glabra*, *Lupinus* sp., and *Lotus pedunculatus*. Lupiwighteone has shown anticancer activity in various cancer cells of neuroblastoma, prostate, and breast cancer [91,92]. It could also induce caspase-dependent and independent apoptosis in breast cancer cells by inhibiting the PI3K/Akt/mTOR pathway [92].

### 3.8. Apigenin

Flavone, apigenin is an active plant-originated compound found in parsley, celery, and chamomile. It has shown to inhibit cancer progression and development by blocking inhibitory-κB kinase (IKK) alpha activation and the PI3K/Akt/FoxO pathway in a TRAMP mice model [125,126]. It also inhibits cell proliferation and induces autophagy by blocking the PI3K/Akt/mTOR pathway in liver cancer cells [127].

### 3.9. Nobiletin

Nobiletin (5,6,7,8,3′,4′-hexamethoxyflavone) is a polymethoxy flavonoid compound derived from citrus fruits [128]. It has shown several pharmacological activities, including anti-oxidative, anti-inflammatory, anticancer, cardio/neuro-protective, and anti-metabolic [128,129]. It has been reported to inhibit ovarian cancer cell growth by inhibiting the secretion of the primary angiogenesis mediators, Akt, hypoxia-inducible factor 1-alpha (HIF-1α), nuclear factor kappa-light-chain-enhancer of activated B cells (NF-κB), and vascular endothelial growth factor (VEGF). Moreover, it does not affect the viability of normal ovarian epithelial cells at less than 40 µM [130].

### 3.10. Galangin

Galangin is a natural flavonoid obtained from honey and *Alpinia officinarum Hance* (*Zingiberaceae*), one of the Chinese herbal medicines. It has various beneficial properties, such as antidiabetic, anticancer, antiviral, and antimicrobial, and does not show any complications [131]. A study reported that galangin could inhibit the proliferation, migration, and invasion of the A498 cells of kidney cancer. Furthermore, it could also induce apoptosis and suppress the PI3K/Akt/mTOR signaling pathway [96].

### 3.11. Hesperidin

Hesperidin is a dietary flavanone widely distributed in citrus fruits, such as oranges, lemon, and lime. Data obtained from several in vitro and in vivo studies suggested a wide spectrum of biological properties associated with hesperidin, which include anti-carcinogenic, antioxidant, and anti-inflammatory [97]. Scientific evidence has indicated that hesperidin induces apoptosis and cell cycle arrest and inhibits cancer cell proliferation by interacting with various cellular targets [31]. Further, it inhibits tumor metastasis, angiogenesis, and chemoresistance [31]. One study reported that hesperidin treatment could induce apoptosis and trigger autophagy by inhibiting the aurora-a mediated PI3K/Akt/mTOR and glycogen synthase kinase 3 beta (GSK-3β) pathway in colon cancer mouse model [132].

### 3.12. Anthocyanins

Anthocyanins are a subclass of flavonoids widely distributed in fruits, such as cherries, berries, grapes, and vegetables, as glycosides, attached to different sugars [133]. Cyanidin is one of the members of the anthocyanin family, which is reported to inhibit cell migration and reverse oxaliplatin-induced EMT biomarker changes through inactivation of PI3K/Akt signaling in hepatocellular carcinoma [100]. Pelargonidin is another member of anthocyanins, and exerts an anticancer effect in human osteosarcoma cells. This anthocyanin’s family member induces autophagy, triggers the ROS induced reduction in mitochondrial membrane potential, and induces cell cycle arrest at the G2/M phase. It also inhibits the expression of p-PI3K and p-Akt in a dose-dependent manner [38].

### 3.13. Delphinidin

Delphinidin plays a vital role in preventing oxidative stress, inflammation, angiogenesis, metastasis, and carcinogenesis [134,135] in different cancers, such as breast [136], prostate [137], lungs [138], liver [139], colon [140], and fibrosarcoma [141] by regulating different cell signal transduction pathways. Delphinidin has shown anti-proliferative properties through inactivation of the PI3K/Akt and ERK1/2 MAPK signaling pathway in ovarian cancer cells [99]. The dose-dependent treatment of delphinidin reduce the SKOV3 cell proliferation by inhibiting the PI3K/Akt and ERK1/2 mitogen-activated protein kinase signaling pathway [99].

### 3.14. Sulforaphane

Sulforaphane is an isothiocyanate, commonly found in cruciferous vegetables. It also possesses anticancer properties and acts as an effective natural agent to modulate the PI3K/Akt signaling pathway. One study demonstrated that sulforaphane inhibits lung cancer cell growth by inhibiting Akt phosphorylation and reduces PTEN expression in lung cancer xenografts mice. Due to this property, sulforaphane could be considered as an important anticancer agent for lung cancer treatment [142].

## 4. Biodisponibility/Bioavailability of Flavonoids

It is well known that human beings have been consuming flavonoids since ancient times. In the modern world, these bioactive flavonoids are widely consumed as part of the diet or nutritional supplements [143,144]. However, low/limited biodisponibility has been an issue that significantly limits the clinical usage of these compounds as anticancer agents [145,146,147]. The poor bioavailability of these flavonoids is due to metabolism carried out by phase II enzymes, resulting in hydrophilic excretable conjugates. Failed or inefficient excretion of these metabolites could hurt overall cellular metabolism, leading to higher exposure to flavonoids [148,149]. To increase the biodisponibility of these flavonoids, the scientific community is focusing their research on limiting the metabolism or targeted delivery of these compounds. These approaches, if successfully implemented, could lead to potent utilization of flavonoids as anticancer agents.

## 5. Conclusions

The above-mentioned scientific literature indicates the role of different signaling pathways in the progression of various cancers. The PI3K/Akt/mTOR is a well-known “hot spot” target for anticancer compounds. Due to natural resources, cost-effectiveness, and ease of use, flavonoids are recommended as anticancer agents. However, even with significant pharmacological potential, they are not fully exploited clinically because of their inherent properties, such as limited bioavailability, rapid metabolism, untargeted delivery, cytotoxicity to normal cells, etc. To enhance their anticancer potential, the possible usage of a mixture of flavonoids has been suggested, considering the probability of affecting different signaling cascades simultaneously. The use of state-of-the-art techniques, including various nanotechnology-based approaches, is also recommended to reduce/nullify the above-listed drawbacks. Their use, alongside currently available chemotherapeutic drugs, could help with reducing required doses, ultimately resulting in fewer side effects.

## Figures and Tables

**Table 1:** Major dietary sources of different flavonoids inhibiting PI3K/Akt/mTOR pathway.

Class of Flavonoids	Inhibitors of PI3K/Akt/mTOR	Dietary Sources	References
**Flavonols**	Quercetin, myricetin, kaempferol, isorhamnetin, ampelopsin	Green tea, black tea, onion, apple with peel, oranges, blueberries, raw spinach, kale, broccoli, almonds, walnuts, dark chocolate, white wine, and red wine	[25,26,27,28,29]
**Flavanol**	EGCG	Green tea, black tea, cranberries, strawberries, red wine, almonds, hazelnuts, and dark chocolate	[30]
**Flavanones**	Hesperidin	citrus fruit, oranges, lemon, and grapefruit	[31]
**Flavones**	Baicalein, acacetin, genkwanin, oroxylin A, pectolinarigenin, galangin	Orange, yellow fruits, spices, and vegetables	[32,33,34]
**Isoflavones**	Genistein, lupiwighteone	Soy, tofu, legumes, *Glycyrrhiza glabra,* Lupinus, and *Lotus pedunculatus*	[35]
**Flavonolignan**	Silibinin	Milk thistle (*Silybum marianum*)	[36]
**Anthocyanins**	Delphinidin, cyanidin, pelargonidin	Red to purplish, blue-colored leafy vegetables, blueberries, other berries, currants, grapes, pomegranate, blue corn, grains, roots, tubers, and red wine	[37,38]

**Table 2:** Different flavonoids and their PI3K/Akt/mTOR inhibitory activity.

Name of Inhibitor	Structure of Inhibitor	Inhibitory Activity	Cancer/Cell Type	Reference
Quercetin	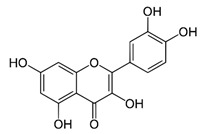	Triggers apoptosis by stimulating autophagy, inhibits the Akt/mTOR pathway	HCC	[26,27]
Myricetin	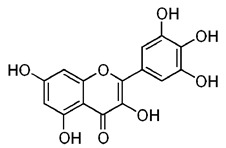	Suppresses angiogenesis by inducing apoptosis, inhibits the PI3K/Akt/mTOR pathway	Endothelial cells	[28]
Kaempferol	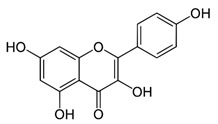	Induces apoptosis, cell cycle arrest at G2/M, inhibits cell migration, PI3k/Akt/mTOR downregulation	Melanoma and liver cancer	[29,85]
Isorhamnetin	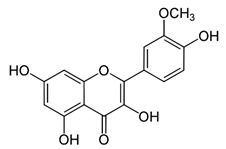	Cell cycle arrest at G2/M phase, inhibits cell proliferation by suppressing PI3K/Akt/mTOR pathway	Colorectal and breast cancer	[33,86]
Ampelopsin	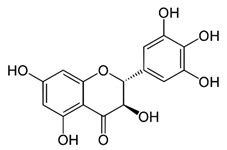	Induces apoptotic and autophagy through the Akt-mTOR pathway via ER stress	MDA-MB-231 and MCF-7 breast cancer	[87]
EGCG	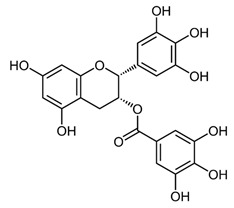	Suppresses the proliferation and induces apoptosis, downregulates the expression of pAkt and p-mTOR, inhibits the PI3K/Akt/mTOR	Gastric carcinoma	[30,88]
Genistein	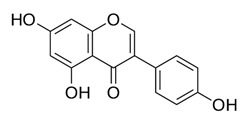	Suppresses Akt and the NF-*κ*B pathway through different cascades	HCC	[89,90]
Lupiwighteone	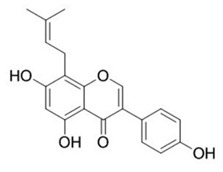	Induces the antiangiogenic activities, triggers the caspase-dependent and independent apoptosis through PI3K/Akt/mTOR pathway inhibition	Prostate and breast cancer	[91,92]
Baicalein	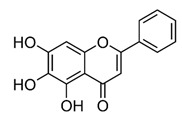	Deactivate PI3K/Akt pathway	HCC	[32]
Acacetin	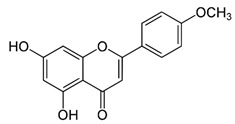	Induces cell cycle arrest at G2/M phase, induces apoptosis and autophagy by suppressing the PI3K/Akt/mTOR pathway	Breast cancer	[33]
Genkwanin	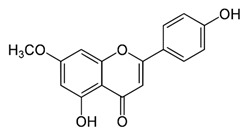	Significantly inhibits cell proliferation, induces cell cycle arrest at G2/M phase, induces apoptosis and autophagy by suppressing the PI3K/Akt/mTOR pathway	Colorectal and breast cancer	[33,34]
Oroxylin A	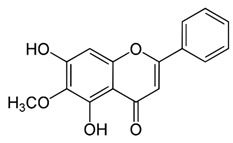	Inhibits the proliferation by inducing autophagy and suppresses the Akt and ERK activation and the phosphorylation of mTOR and STAT3	Glioma cells	[93,94]
Pectolinarigenin	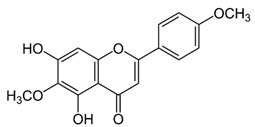	Induces cell cycle arrest at G2/M phase, induces autophagic and apoptotic cell death through downregulation of PI3K/Akt/mTOR pathway	Gastric cancer	[95]
Galangin	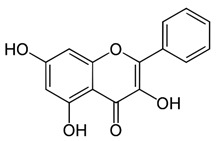	Inhibits the cell proliferation, migration and invasion, induces apoptosis, suppresses PI3K/Akt/mTOR signaling	A498 cells	[96]
Hesperidin	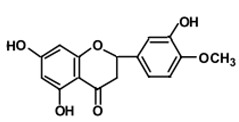	Induces apoptosis and autophagy by inhibiting Aurora-A mediated PI3K/Akt/mTOR and GSK-3β pathways	Colon cancer	[31,97]
Silibinin	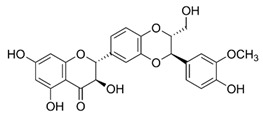	Anti-proliferative, inhibits HIF-1*α* and the mTOR/p70S6K/4E-BP1 signaling pathway	Hepatoma cells	[36,98]
Delphinidin	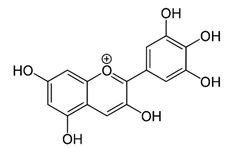	Inhibits cell proliferation by inactivating the PI3K/Akt, and ERK1/2 mitogen-activated protein pathway	Ovarian cancer	[99]
Cyanidin	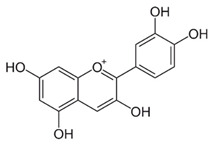	Inhibits cell migration and reverses drug resistance by suppressing the PI3K/Akt pathway	HCC	[100]
Pelargonidin	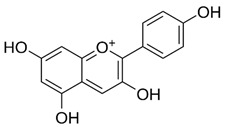	Triggers autophagy and ROS-induced decline in MMP, cell cycle arrest at the G2/M phase through inhibiting PI3K and p-Akt signaling	Human osteosarcoma	[38]

HCC, hepatocellular carcinoma; MMP, mitochondrial membrane potential.

## Data Availability

Not applicable.

## Abbreviations

Akt	Protein kinase
BAD	BCL2 associated agonist of cell death
BCL2	B-cell lymphoma 2
4EBP1	Eukaryotic translation initiation factor 4E binding protein 1
EGCG	Epigallocatechin gallate
EGFR	Epidermal growth factor receptor
eIF4E	Eukaryotic translation initiation factor 4E
ERK	Extracellular signal-regulated kinase
GPCRs	G-protein-coupled receptors
GSK-3β	Glycogen synthase kinase 3 beta
GLUT4	Glucose transporter type 4
HER2	Human epidermal growth factor receptor 2
HIF-1α	Hypoxia-inducible factor 1-alpha
IKK	Inhibitory-κB kinase
MAPK	Mitogen-activated protein kinase
MEK MITF	Mitogen-activated protein kinase kinase Microphthalmia-associated transcription factor
NF-κB NO	Nuclear factor kappa-light-chain-enhancer of activated B cells Nitric oxide
mTOR	mammalian target of rapamycin
PI3K	Phosphoinositide 3-kinase
PH	Pleckstrin homology
PDK1	Phosphoinositide-dependent kinase-1
p70S6K1	p70 ribosomal S6 kinase 1
PIK3CA	Phosphatidylinositol 4,5-bisphosphate 3-kinase catalytic subunit alpha
PIP3	Phosphatidylinositol-3,4,5-triphosphate
PP2A	Protein phosphatase 2A
PTEN	Phosphatase and tensin homolog deleted on chromosome 10
RHEB GDP	Ras homolog enriched in brain GDP
RHEB GTP	Ras homolog enriched in brain GTP
ROS	Reactive oxygen species
RTK	Receptor tyrosine kinase
S6k1	Ribosomal protein S6 kinase beta-1
TSC	Tuberous sclerosis complex
VEGF	Vascular endothelial growth factor
